# Distinctive Distribution of Secretory Phospholipases A_2_ in the Venoms of Afro-Asian Cobras (Subgenus: *Naja*, *Afronaja*, *Boulengerina* and *Uraeus*)

**DOI:** 10.3390/toxins11020116

**Published:** 2019-02-14

**Authors:** Choo Hock Tan, Kin Ying Wong, Nget Hong Tan, Tzu Shan Ng, Kae Yi Tan

**Affiliations:** 1 Department of Pharmacology, Faculty of Medicine, University of Malaya, Kuala Lumpur 50603, Malaysia; tanch@um.edu.my (C.H.T.); kinying12@gmail.com (K.Y.W.); 2 Department of Molecular Medicine, Faculty of Medicine, University of Malaya, Kuala Lumpur 50603, Malaysia; tanngethong@yahoo.com.sg (N.H.T.); ngtzushan@um.edu.my (T.S.N.)

**Keywords:** svPLA_2_, acidimetric assay, colorimetric assay, venom enzymatics, immunoreactivity, cobra venom

## Abstract

The protein abundances of phospholipases A_2_ in cobra venom proteomes appear to vary among cobra species. To determine the unique distribution of snake venom phospholipases A_2_ (svPLA_2_) in the cobras, the svPLA_2_ activities for 15 cobra species were examined with an acidimetric and a colorimetric assay, using egg yolk suspension and 4-nitro-3-octanoyloxy benzoic acid (NOBA) as the substrate. The colorimetric assay showed significant correlation between svPLA_2_ enzymatic activities with the svPLA_2_ protein abundances in venoms. High svPLA_2_ activities were observed in the venoms of Asiatic spitting cobras (*Naja sputatrix*, *Naja sumatrana*) and moderate activities in Asiatic non-spitters (*Naja naja*, *Naja atra*, *Naja kaouthia*), African spitters (subgenus *Afronaja*), and forest cobra (subgenus *Boulengerina*). African non-spitting cobras of subgenus *Uraeus* (*Naja haje*, *Naja annulifera*, *Naja nivea*, *Naja senegalensis*) showed exceptionally low svPLA_2_ enzymatic activities. The negligible PLA_2_ activity in *Uraeus* cobra venoms implies that PLA_2_ may not be ubiquitous in all snake venoms. The svPLA_2_ in cobra envenoming varies depending on the cobra species. This may potentially influence the efficacy of cobra antivenom in specific use for venom neutralization.

## 1. Introduction

Phospholipases A_2_ (PLA_2_) (EC 3.1.1.4) are enzymes that hydrolyze glycerophospholipids to lysophospholipids and fatty acids. The first snake venom PLA_2_ enzymes were purified from the venoms of *Naja naja* and *Naja tripudians* as hemolysin due to their ability to lyse the phospholipid membranes of red blood cells [1]. Since then, various snake venom-derived PLA_2_ (svPLA_2_) have been characterized and shown to exist in virtually all venoms from the two major families of venomous snakes: Elapidae and Viperidae [2]. Homologous svPLA_2_ are especially abundant and diverse in the Asiatic elapids, including cobras, coral snakes, kraits, and some sea snake species [3,4,5,6], implying that the enzyme plays an essential role in the function of the venom.

Previous studies demonstrated that svPLA_2_ originated from ancestral physiological genes that have subsequently undergone several convergent and divergent evolutionary events crucial for the adaptation and survival of the snakes [7]. Typically, the snake venom PLA_2_ are single-chain polypeptides with 115–125 amino acid residues (13–15 kDa), and high degrees of sequence homology are observed across different cobra species [8]. Despite sequence similarity, svPLA_2_ can differ widely in their pharmacology, contributing to the diverse toxic activities in snakebite envenoming. In the pathophysiology of elapid snake envenoming, the svPLA_2_ are commonly associated with presynaptic neurotoxicity (kraits [9,10]), myotoxicity (sea snakes [3,11,12]) (suggest to remove citation 3 as the article do not contain information of sea snake myotoxicity) and possibly cardiotoxicity (king cobra [13]). In certain species of Asian cobras, such as the Javan spitting cobra (*Naja sputatrix*) and the equatorial spitting cobra (*Naja sumatrana*), the basic and neutral svPLA_2_ isoenzymes are known to contribute substantially to lethality in mice, with or without a synergistic action with the cobra venom cytotoxins/cardiotoxins [3,14,15]. Venom proteome data reported to date revealed that the PLA_2_ account for approximately 4−31% of venom protein content of various cobra species including *Naja naja* [16,17,18,19], *Naja sputatrix* [14], *Naja atra* [20,21], *Naja kaouthia* [20,22,23,24], *Naja siamensis* [20], *Naja melanoleuca* [25], *Naja nigricollis*, *Naja katiensis*, *Naja pallida*, *Naja nubiae*, *Naja mossambica* [26], *Naja ashei* [27], and *Naja haje* [28]. This indicates that svPLA_2_ is an important component in cobra venoms and likely plays an important role in envenoming of human, in addition to predatory and/or digestive functions. However, interspecific differences in the svPLA_2_ enzymatic activities of some cobra species had been reported. Particularly noteworthy is the extremely low level of PLA_2_ activity in two African cobra species, *Naja nivea* [29,30] and *Naja haje*, in comparison to several other Asian and African cobra species [30]. As such, the difference in the svPLA_2_ enzymatic activities could be a potential indicator for distinguishing the cobra venoms from different evolutionary clades of cobras.

Despite the findings reported decades ago, the taxonomic value of this information pertaining to the measurement of PLA_2_ enzymatic activities had since remained under-explored. This is a relevant research topic to be revisited, especially when the *Naja* cobra complex has undergone several taxonomic revisions since then. The *Naja* cobras are now known to include four subgenera: *Naja*, *Afronaja*, *Boulengerina,* and *Uraeus* [31,32]. The subgenus *Naja* represents the Asiatic lineage of both spitting and non-spitting cobras. The other three subgenera include the African species, where the spitting cobras are grouped under the subgenus *Afronaja*, whereas the non-spitting cobras are grouped under the subgenera *Boulengerina* and *Uraeus*. In total, there are no less than 20 distinct species under the genus *Naja* currently, compared to what were only recognized as six species half a century ago [32,33]. Thus, the profiling of PLA_2_ distribution in the vast cobra venoms is worthy of investigation to obtain valuable insights into the natural history of venom evolution. The knowledge gained is also important to increase the understanding of cobra venom toxicity in the context of the biogeography and phylogeny of cobras. In this study, we investigated the svPLA_2_ enzymatic activities for various cobra species from different geographical locales using two independent enzymatic assays. The findings were analyzed in correlation to the protein abundances of the PLA_2_ enzyme reported in various cobra venoms.

## 2. Results

### 2.1. PLA_2_ Enzymatic Activities (Acidimetric Assay)

**Figure 1:**
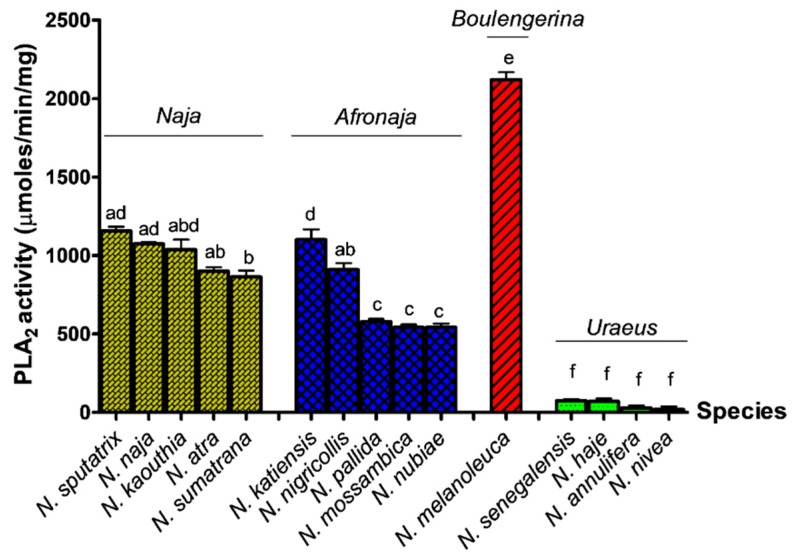
Comparison of venom phospholipase A_2_ activities in acidimetric assay among the venoms of four subgenera of cobra. Values are expressed as mean ± S.E.M. of triplicates. Statistical analysis difference was performed by one-way analysis of variance (ANOVA) and Tukey’s post hoc test, where the statistical significance (*p* < 0.05) is indicated by different lower-case letters at the top of the bars. Bars without any common lowercase letter denote values that are significantly different (*p* < 0.05).

The pH of substrate (egg yolk suspension) generally reduced with time when reacting with the cobra venoms (Figure S1). The highest PLA_2_ activity in the acidimetric assay was noted in *N. melanoleuca* venom (subgenus *Boulengerina*) (rate = 2120.66 µmol/min/mg), followed by the venoms of Asian cobras (subgenus *Naja*) (rates = 864.04–1157.56 µmol/min/mg) and African spitting cobras (subgenus *Afronaja*) (rates = 543.01–1102.52 µmol/min/mg). The venoms of African non-spitting cobras (subgenus *Uraeus*) showed extremely low PLA_2_ enzymatic activity (rates = 20.18–75.21 µmol/min/mg). Figure 1 illustrates the PLA_2_ enzymatic activities of cobra venoms by subgenus tested in the acidimetric assay.

### 2.2. PLA_2_ Enzymatic Activities (Colorimetric Assay)

**Figure 2:**
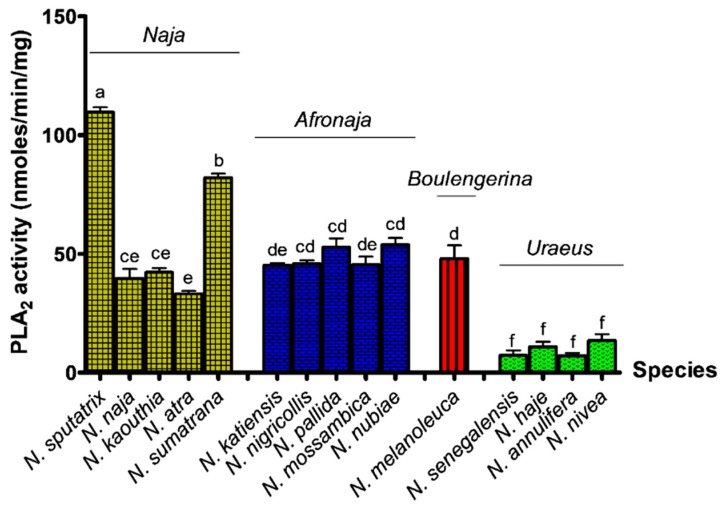
Comparison of venom phospholipase A_2_ (PLA_2_) activities in colorimetric assay for the venoms of four subgenera of cobra. Values are expressed as mean ± S.E.M. of triplicates. Statistical analysis difference was performed by one-way ANOVA and Tukey’s post hoc test, where the statistical significance (*p* < 0.05) is indicated by different lower-case letters at the top of the bar. Bars without any common lowercase letter denote values that were significantly different (*p* < 0.05).

The PLA_2_ activities of the cobra venoms were measured using a colorimetric assay. The enzymatic activity of the venoms in hydrolyzing the non-micellar substrate (NOBA) over time is shown in Figure S2. High PLA_2_ activities were noted in the venoms of *N. sputatrix* (rate = 109.69 nmol/min/mg) and *N. sumatrana* (rate = 82.11 nmol/min/mg), followed by other species in the subgenera *Naja* (rate = 33.21–42.26 nmol/min/mg), *Afronaja* (rate = 45.15–53.82 nmol/min/g), and *Boulengerina* (rate = 48.03 nmol/min/mg). In comparison, cobra venoms of the *Uraeus* subgenus showed much lower PLA_2_ activities (rate = 7.12–13.52 nmol/min/mg) (Figure 2).

### 2.3. Correlation Between PLA_2_ Activities and PLA_2_ Abundances in Cobra Venoms

**Figure 3:**
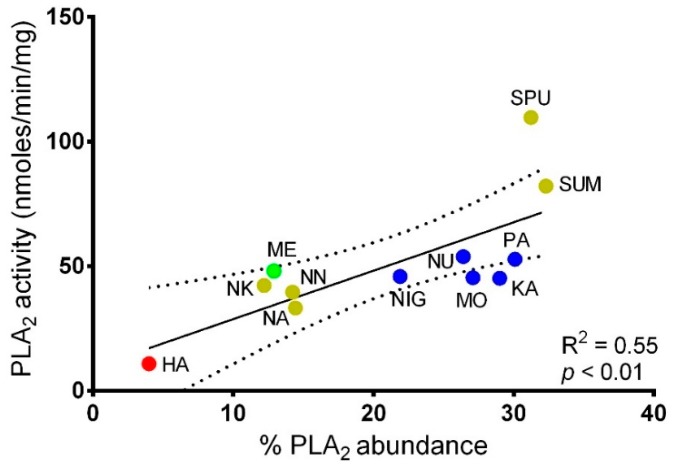
Correlation plot of PLA_2_ enzymatic activities and PLA_2_ protein abundances in cobra venoms. Enzymatic measurement using colorimetric assay for 12 cobra venoms. The relative abundance of PLA_2_ (% by total venom proteins) in 12 cobra venoms were adopted from published literature for subgenus *Naja*: *N. sputatrix* [14], *N. naja* [19], *Naja atra* [20], *N. kaouthia* [22], *Naja sumatrana* [34]; subgenus *Afronaja*: *N. katiensis*, *N. mossambica*, *N. pallida*, *N. nubiae,* and *N. nigricollis* [26]; subgenus *Boulengerina*: *N. melanoleuca* [25]; and subgenus *Uraeus*: *N. haje* [28]. Abbreviations: *R*^2^: Coefficient of determination; NA, *N. atra*; NK, *N. kaouthia*; NN, *N. naja*; SUM, *N. sumatrana*; SPU, *N. sputatrix*; NIG, *N. nigricollis*; KA, *N. katiensis*; NU, *N. nubiae*; MO, *N. mossambica*; PA, *N. pallida*; ME, *N. melanoleuca*; HA, *N. haje*.

Figure 3 shows the correlation between PLA_2_ activities in the colorimetric assay and PLA_2_ protein abundances in cobra venoms. The PLA_2_ protein abundances of 12 cobra species were obtained from published studies that adopted a comparable quantitative approach, in which the protein abundances were estimated based on peak areas of reverse-phase high performance liquid chromatography (HPLC), followed by integration with the relative mass spectral intensity or relative gel band density of PLA_2_ eluted (Table S1) [14,19,20,22,25,26,28,34]. The PLA_2_ activities measured using the acidimetric assay showed a weak correlation with the PLA_2_ abundances (coefficient of determination, *R*^2^ = 0.01, *p* > 0.05). The colorimetric assay demonstrated a moderate to strong association between the PLA_2_ activities and PLA_2_ abundances of the cobra venoms studied (*R*^2^ = 0.55, *p* < 0.01) (Figure 3). The higher PLA_2_ enzymatic activities were observed in the venoms of spitting cobras under the subgenus *Naja* (*N. sputatrix* and *N. sumatrana*) and subgenus *Afronaja* (African spitters), whose PLA_2_ abundances were more than 20% of total venom proteins. The non-spitting Asiatic *Naja* cobras (*N. kaouthia*, *N. naja* and *N. atra*) had intermediate PLA_2_ abundances (12–14%) with moderate PLA_2_ enzymatic activities per unit venom. The venom of *N. haje* (African non-spitting cobra under the *Uraeus* subgenus), however, showed very low PLA_2_ enzymatic activity that was in line with its low PLA_2_ content (4%).

Figure 3 depicts the correlation between PLA_2_ activities exhibited by the whole venoms and relative abundances of PLA_2_ in the venoms. The PLA_2_ activities were measured using whole venoms and are expressed in nmol/min/mg venom proteins as previously established [29,35]. This allowed the determination of correlation between the PLA_2_ activities (measured per unit mass of venom) and the relative abundances of PLA_2_ in the cobra venoms. The higher the PLA_2_ relative abundance (% of total venom proteins) in a venom, the higher the snake venom PLA_2_ activity. The PLA_2_ activity of snake venom can also be expressed as “PLA_2_ specific activity” [36], where the PLA_2_ activity of a whole venom is normalized (divided) by the amount of PLA_2_ in the venom. This measurement is suitable for characterizing the enzymatic activity of purified or isolated PLA_2_, expressed in the unit of nmol/min/mg PLA_2_ isolated [36]. In this study, the PLA_2_ specific activity is included along with the relevant parameters used in calculating the values (Table 1). The findings showed that the cobra venoms tested could be generally classified into four groups: (1) Asiatic spitting cobras under the subgenus *Naja* (*N. sputatrix* and *N. sumatrana*), showing higher PLA_2_ activity with higher venom PLA_2_ abundance (>30%) and higher PLA_2_ specific activity (254–351 nmol/min/mg PLA_2_ protein); (2) African spitting cobras (subgenus *Afronaja*), showing intermediate PLA_2_ activities with high PLA_2_ abundances (>20%) and lower PLA_2_ specific activities (156–209 nmol/min/mg PLA_2_ protein); (3) non-spitting Asiatic *Naja* cobras (*N. kaouthia*, *N. naja* and *N. atra*) and African forest cobra *N. melanoleuca* (*Boulengerina*), showing intermediate PLA_2_ activities with moderate PLA_2_ abundances (12–14%) and higher specific activities (230–372 nmol/min/mg PLA_2_ protein); and (4) *N. haje* (African non-spitting cobra under the *Uraeus* subgenus), showing very low PLA_2_ enzymatic activity with low PLA_2_ content (4%) and higher specific activities (272 nmol/min/mg PLA_2_ protein) (Table 1).

### 2.4. Phylogenetics of Cobras in Relation to Venom PLA_2_ Activities

**Figure 4:**
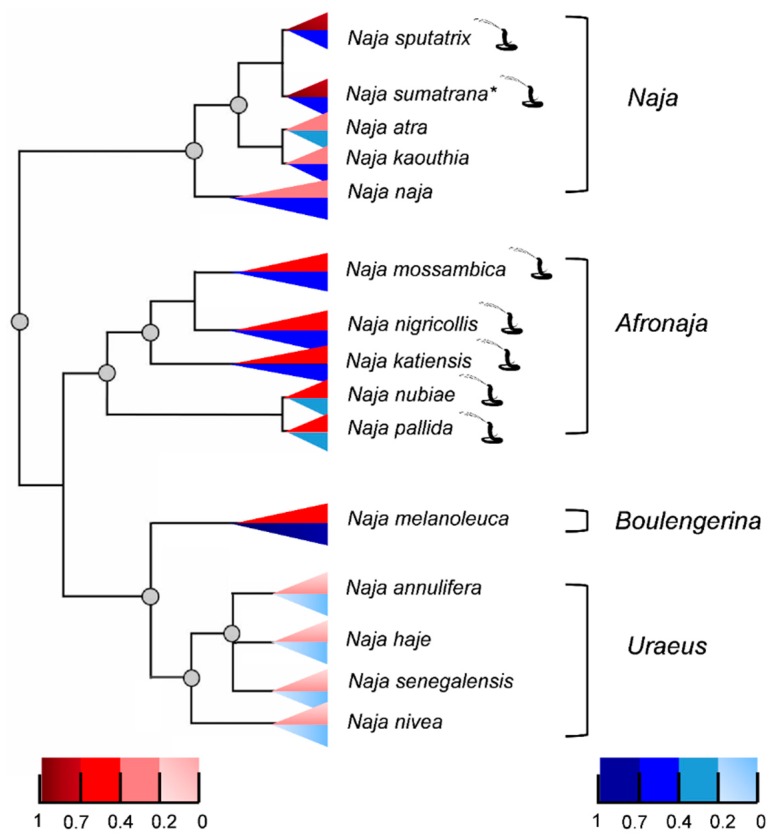
Phylogenic tree relating the venom phospholipase A_2_ activities of 15 cobra species by subgenera. Blue: acidimetric assay; red: colorimetic assay. The venom PLA_2_ activity is expressed in a ratio relative to the highest activity detected by acidimetric or colorimetric assay in this study (1.0 implies the highest activity). More intense color indicates higher PLA_2_ activity. The phylogenetic tree was redrawn with adaptation from phylogenetics of cobras [32]. *Naja sumatrana* is depicted here as a sister taxon of *Naja sputatrix*. Spitting cobras are marked with the snake symbol next to their species name.

The relative PLA_2_ enzymatic activities of the 15 cobra venoms (by acidimetric and colorimetric methods) are related to the phylogeny of cobras in Figure 4. The venoms of Asian spitting cobras (*N. sputatrix* and *N. sumatrana*) exhibited the highest PLA_2_ enzymatic activity tested with the colorimetric method, whereas the highest PLA_2_ activity determined by the acidimetric method was observed in the African forest cobra *N. melanoleuca* venom. The venoms of other cobra species within the subgenera *Naja* (Asian cobras) and *Afronaja* (African spitting cobras) showed moderate to high levels of PLA_2_ activities in both enzymatic assays. The venoms of cobras within the *Uraeus* subgenus, representing a monophyletic group of non-spitting African cobras separated from the African forest cobras more recently, showed exceptionally low PLA_2_ activities in both acidimetric and colorimetric assays.

## 3. Discussion

Phospholipase A_2_ (phosphatidylcholine 2-acylhydrolase) catalyzes the hydrolysis of phosphatidylcholine at the sn-2 ester bond to produce lysophospholipid and free fatty acids. We tested the activities of svPLA_2_ based on two different types of PLA_2_ assays [37]: an acidimetric assay that measured the release of proton from fatty acids during the hydrolysis of phosphate ester bond, and a colorimetric assay that measured the amount of chromogenic 4-nitro-3-hydroxybenzoic acid released by the cleavage of PLA_2_ at the ester bond between the octanol group of NOBA [38]. The activities tested on NOBA showed a better correlation with the PLA_2_ contents in the cobra venoms. The egg-yolk-based acidimetric assay probably contained less specific substrates (phospholipids, triglycerides) that could be targets of other lytic enzymes in the venoms, or atmospheric carbon dioxide could have interfered with the assay by dissolving into the suspension during the stirring process. On the whole, the enzymatic rates measured by both assays support that PLA_2_ enzymatic activities vary according to the svPLA_2_ composition in different cobra species. The enzymatic svPLA_2_ distribution is unique following a clustering trend among the four subgenera, and notably the remarkable lack of svPLA_2_ within the *Uraeus* subgenus.

The enzymatic activities measured in this study for the venoms of African spitting cobras (*N. nubiae*, *N. nigricollis*, *N. mossambica*, *N. pallida*, *N. katiensis*) and those from Asia (*N. sumatrana*, *N. sputatrix*) were high, consistent with the high abundances of svPLA_2_ reported previously in these venoms [14,26,34]. Some svPLA_2_ of *Afronaja* cobras were shown to be cytotoxic and to have dermonecrotic and myonecrotic activities [39,40]. Some African spitting cobra venoms possess coagulotoxic activity, which were inhibited by the use of phospholipase A_2_ inhibitor LY315920 *in vitro* [41]. In envenoming, bites from the African spitting cobras (*Afronaja*) are commonly associated with local tissue damages [42,43], which could be attributed to the svPLA_2_ and cytotoxins present abundantly in these venoms [26]. Previous studies also indicated that cobra svPLA_2_ enzymes worked synergistically with cytotoxins (cardiotoxins) to enhance venom toxicity [44,45] and the combination are probably responsible for causing venom ophthalmia (venom-induced conjunctivitis, chemosis, corneal erosions). Although the Asiatic non-spitters (*N. naja*, *N. kaouthia*, *N. atra*) also showed remarkably high PLA_2_ activities (correlated with the composition); their svPLA_2_ are mainly of acidic subtypes that lack lethal activity [46,47]. Two acidic PLA_2_ subtypes from Indian *N. kaouthia* venom were reported previously to exhibit anticoagulant activity [48]; this coagulopathic or hemotoxic effect, however, has not been commonly reported in clinical cobra envenoming. Similarly, the African forest cobra *(N. melanoleuca*, subgenus *Boulengerina*) is a non-spitting cobra species whose venom exhibited a strong PLA_2_ activity in this study; however, its svPLA_2_ had been shown to play no crucial role in the toxicity of the venom [25]. The pathophysiological role of these apparently non-toxic svPLA_2_ of cobras remain to be further elucidated, although these enzymes probably have more important ecological roles for the adaptation of the cobras to different niches. The lack of svPLA_2_ in the African non-spitting cobras of the subgenus *Uraeus* is a unique venom phenotype unveiled in this study. The finding implies that the svPLA_2_ is probably the least medically significant in the envenoming by this group of African cobras.

Snake venom PLA_2_s are isoenzyme products of multiple genes, and are further divided into distinct groups based on the differences in the number of disulfide bonds and the presence/absence of an N-terminal heptapeptide [49]. The svPLA_2_ of elapid snakes (including cobras) belongs to Group Ia PLA_2_ isoenzymes, which are homologous with the mammalian pancreatic PLA_2_ (Group Ib PLA_2_). Lynch [50] concluded that in Group I PLA_2_ enzymes, gene duplication and diversification occurred after speciation. This implies that the sequence homology and antigenicity of cobra svPLA_2_ are probably divergent between the different species of an individual subgenus. Hence, an antibody used against a PLA_2_ subtype of a specific cobra species may reveal variable immunoreactivity between different cobra venoms. From a practical point of view, the potentially diverse svPLA_2_ antigenicity and varying svPLA_2_ protein abundance among the different cobras pose challenges for antivenom production and usage in some regions. The phenomenon may variably affect the neutralizing efficacy of antivenoms that are produced and used for specific treatment against the envenoming by different cobra species in Asia and Africa [51,52].

From the phylogenetic perspective, the moderate-to-high enzymatic activity of svPLA_2_ is common in the *Afronaja*, *Naja* and *Boulengerina* lineages. Within the Asiatic *Naja* subgenus, the high PLA_2_ enzymatic activities along with the emergence of basic and neutral svPLA_2_ which are lethal, and the ability to ‘spit’ (to be exact, spray) venom, represents a more recently derived venom phenotype and defense trait unique to some of the Asiatic spitting cobras (*N. sputatrix* and *N. sumatrana* in this study). Although the Chinese/Formosan cobra (*N. atra*) and an unrecorded subpopulation of the Thai monocled cobra (*N. kaouthia*) were anecdotally reported to spit/spray venom on rare occasions, these two species are not considered accomplished spitters in this study due to their lack of formally documented specialized dental adaptations. The Asiatic spitting cobras, however, are known to be less accurate spitters compared with their African counterparts of the *Afronaja* subgenus that might have evolved a better defensive trait of venom spraying [53,54]. Some spitting cobras, e.g. *N. sumatrana* and *N. sputatrix*, produce lethal basic and/or neutral svPLA_2_ related to the pathophysiology of systemic envenoming and venom ophthalmia [2,3,14,55].

The subgenus *Uraeus* broadly encompasses non-spitting cobras of the *N. haje* complex living in the open areas of Africa. The extremely low svPLA_2_ activities and the negligible PLA_2_ enzyme content are unique venom phenotypes in the *Uraeus* cobras, reflecting a less critical role of svPLA_2_ in envenomation. The negligible svPLA_2_ content could be correlated with weak cytotoxicity and low dermonecrotic activity of the venoms. These venoms are generally more neurotoxic among the African cobras. The loss of svPLA_2_ functions in the lineage probably followed a decelerated mode of evolution or pseudogenization of the svPLA_2_ as the cobras diverged from the common ancestor shared by their closest kin, the forest cobra of *Boulengerina*, which retained or evolved a venom with a high svPLA_2_ enzymatic activity. The underlying cause, mechanism, and implication of the evolutionary event await further investigation.

## 4. Conclusions

The present study demonstrated the correlation of svPLA_2_ enzymatic activities with the enzyme protein abundances in Afro-Asian cobras of different subgenus. The dominant presence of enzymatically active svPLA_2_ is in line with the emergence of venom-spitting (spraying) behavior, once in the African *Naja* (*Afronaja*) spp., and once in the more derived Asiatic spitters of *Naja* (*Naja*) spp. The African non-spitting cobras of the subgenera *Boulengerina* and *Uraeus* diverged with a distinctive svPLA_2_ distribution: the forest-dwelling species (*Boulengerina*) continued to use a venom rich in svPLA_2_, whereas the open-land species (*Uraeus*) adapted to a venom that has little or negligible svPLA_2_. The lack of svPLA_2_ in the venom phenotype of African non-spitters from the *Uraeus* subgenus is hence striking. This provides an alternative view on the commonly perceived ubiquitous presence of svPLA_2_ in cobra venoms, and implies that the significance of svPLA_2_ in cobra envenoming varies among different species.

## 5. Materials and Methods

### 5.1. Consumables and Reagents

All chemicals and reagents used in the studies were of analytical grade. Hydrogen peroxide and sulfuric acid were supplied by J. T. Baker (Phillipsburg, NJ, USA). Goat anti-horse IgG-horseradish peroxidase (HRP) conjugate was supplied by Bio-Rad Laboratories (Hercules, CA, USA). 4-nitro-3-octanoyloxy benzoic acid (NOBA) was supplied by Santa Cruz Biotechnology (Santa Cruz, CA, USA). The 15 cobra venoms (*Naja* species) used in this study were sourced from various localities. Venoms of *Naja naja* (Pakistan), *Naja sputatrix* (Indonesia), *Naja pallida* (Kenya), *Naja katiensis* (Burkina Faso), *Naja mossambica* (South Africa), *Naja nigricollis* (Cameroon), *Naja nubiae* (Egypt), *Naja melanoleuca* (Cameroon), *Naja haje* (Egypt), *Naja annulifera* (Mozambique), *Naja senegalensis* (Mali), and *Naja nivea* (South Africa) were supplied by Latoxan (Valence, France). Venoms of *Naja kaouthia* (Thailand), *Naja sumatrana* (Malaysia), and *Naja atra* (Taiwan) were pooled samples from multiple adult snakes of the respective regions.

### 5.2. PLA_2_ Assay (Acidimetric Method)

Phospholipase A_2_ activities of the cobra venoms were determined by the acidimetric method as described by Tan and Tan [56]. The egg yolk substrate was prepared in a suspension constituted of chicken egg yolk, 18 mM calcium chloride, and 8.1 mM sodium deoxycholate in a 1:1:1 ratio. The substrate suspension pH was adjusted to 8.0 using sodium hydroxide. To ensure good mixing, the substrate suspension was continuously stirred at room temperature. One hundred microliters of venom solution (containing 10 µg venom) was added to 5 mL of the substrate suspension. The rate of pH decrease was recorded using a pH meter. A decrease of 1 pH unit of the egg yolk suspension corresponded to 133 µmol of fatty acids released. The enzyme activity is expressed as µmoles of fatty acids released/min/mg. The values are expressed as means ± S.E.M. of triplicates.

### 5.3. PLA_2_ Assay (Colorimetric Method)

Phospholipase A_2_ activities of the cobra venoms were assayed according to the colorimetric method as described by Holzer and Mackessy [35] and modified for use in a 96-well plate [57] using the synthetic chromogenic substrate (NOBA). Briefly, the standard assay mixture contained 200 µL of buffer (10 mM Tris-HCl, 10 mM CaCl_2_, and 100 mM NaCl, pH 8.0), 20 µL of substrate (3 mM), 20 µL of water, 20 µL of PLA_2_, and a final volume of 260 µL. Sample (10 µg in 20 µL) was then added to the mixture and incubated at 37 °C for 40 min, with the reading of absorbance at kinetic interval of 5 min over a period of 60 min at 425 nm using Tecan i-control™ infinite M1000Pro microplate reader (Männedorf, Switzerland). The enzyme activity, expressed as the initial velocity of the reaction, was determined by calculating the increase in absorbance after 20 min at 425 nm. The enzyme activity is expressed as mean ± S.E.M of triplicates.

### 5.4. Statistical Analyses

The PLA_2_ activities of both acidimetric and colorimetric assays are expressed as means ± S.E.M. (standard error of mean) of triplicates. The statistical differences between the venom samples in individual assay were analyzed by one-way analysis of variance (ANOVA) (*p* < 0.05) followed by Tukey’s post hoc test using SPSS version 20 software (IBM, Armonk, NY, USA, 2016). Lower-case letters are labelled at the top of the bars to indicate if there is a significant difference between the values. Sharing of any common lower-case letters between bars indicates there was no significant difference (*p* > 0.05) between the values charted, whereas bars that do not share any common lowercase letters have values that are significantly different from one another (*p* < 0.05). The relationship between the PLA_2_ activity and the relative abundance of PLA_2_ in each venom sample was also studied by linear regression analysis, using Graphpad Prism 6 software (San Diego, CA, USA). The strength of the association was interpreted by the coefficient of determination (*R*^2^) and the cut-off value at *p* < 0.01 indicates a highly significant regression between the PLA_2_ activity and the PLA_2_ protein abundance.

## Figures and Tables

**Table 1:** Cobra venom PLA_2_ activities and PLA_2_-specific activities.

Subgenus	Cobra Venom	Relative PLA_2_ Abundance (% Total Venom Proteins)	PLA_2_ Activity of Venom (nmol/min/mg Venom Proteins)	PLA_2_ Specific Activity (nmol/min/mg PLA_2_ Protein)
***Naja***	*N. naja*	14.2	39.57	277.88
	*N. kaouthia*	12.2	42.26	346.38
	*N. sputatrix*	31.2	109.70	351.13
	*N. atra*	14.4	33.21	230.12
	*N. sumatrana*	32.3	82.11	254.21
***Afronaja***	*N. nigricollis*	21.9	45.85	209.36
	*N. pallida*	30.1	52.78	175.36
	*N. nubiae*	26.4	53.82	203.84
	*N. mossambica*	27.1	45.35	167.36
	*N. katiensis*	29.0	45.15	155.69
***Boulengerina***	*N. melanoleuca*	12.9	48.03	372.33
***Uraeus***	*N. haje*	4.0	10.87	271.71

## Supplementary Materials

The following are available online at http://www.mdpi.com/2072-6651/11/2/116/s1, Figure S1: Time-dependent pH changes in acidimetric assay for the venoms of four subgenera of cobra: (A) *Naja*, (B) *Afronaja*, (C) *Boulengerina*, and (D) *Uraeus*. Hydrolysis of phospholipids by phospholipase A_2_ released fatty acids that reduced the suspension pH in a time-dependent manner. Figure S2: Time-dependent absorbance changes in colorimetric assay for the venoms of four subgenera of cobra: (A) *Naja*, (B) *Afronaja*, (C) *Boulengerina*, and (D) *Uraeus*. Changes in absorbance were due to the hydrolysis of the synthetic chromogenic substrate (NOBA), corresponding to the enzymatic activity of phospholipases A_2_ in the venoms. Table S1: Relative abundances of snake venom phospholipase A_2_ of 12 cobra species (Genus: *Naja*).
