# Supplementary material for: Distinctive Distribution of Secretory Phospholipases A2 in the Venoms of Afro-Asian Cobras (Subgenus: Naja, Afronaja, Boulengerina and Uraeus)
Source: Toxins (Basel). 2019 Feb 14;11(2):116. doi: 10.3390/toxins11020116 (PMC6410299; doi:10.3390/toxins11020116)
Supplement: Supplementary file 1 [file toxins-11-00116-s001.pdf]

Supplementary Materials: Distribution of Secretory Phospholipases A2 in the Venoms of Afro-Asian Cobras (Subgenus: *Naja*, *Afronaja*, *Boulengerina* and *Uraeus*)

Choo Hock Tan, Kin Ying Wong, Nget Hong Tan, Tzu Shan Ng and Kae Yi Tan

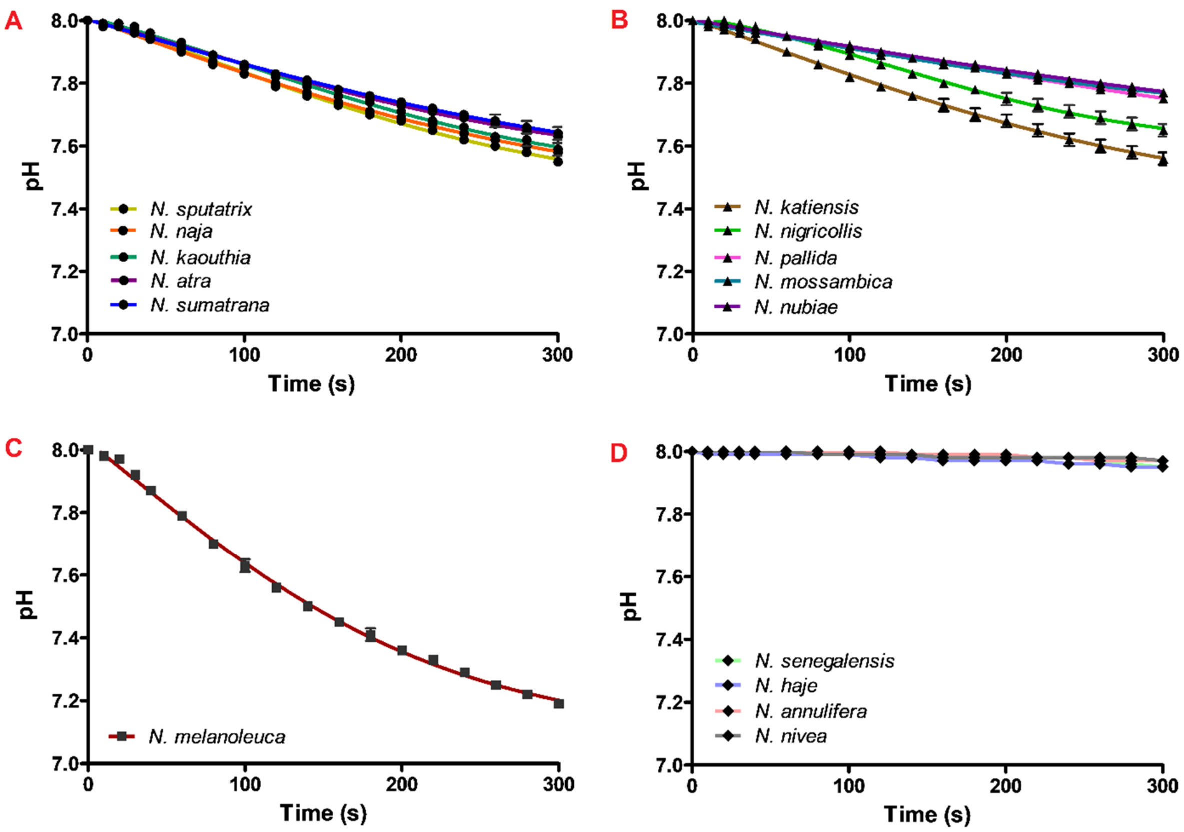

Figure 1. Time-dependent pH changes in acidimetric assay for the venoms of four subgenera of cobra. (**A**) *Naja*, (**B**) *Afronaja*, (**C**) *Boulengerina*, and (**D**) *Uraeus*. Hydrolysis of phospholipids by phospholipase A2 released fatty acids that reduced the suspension pH time-dependently.

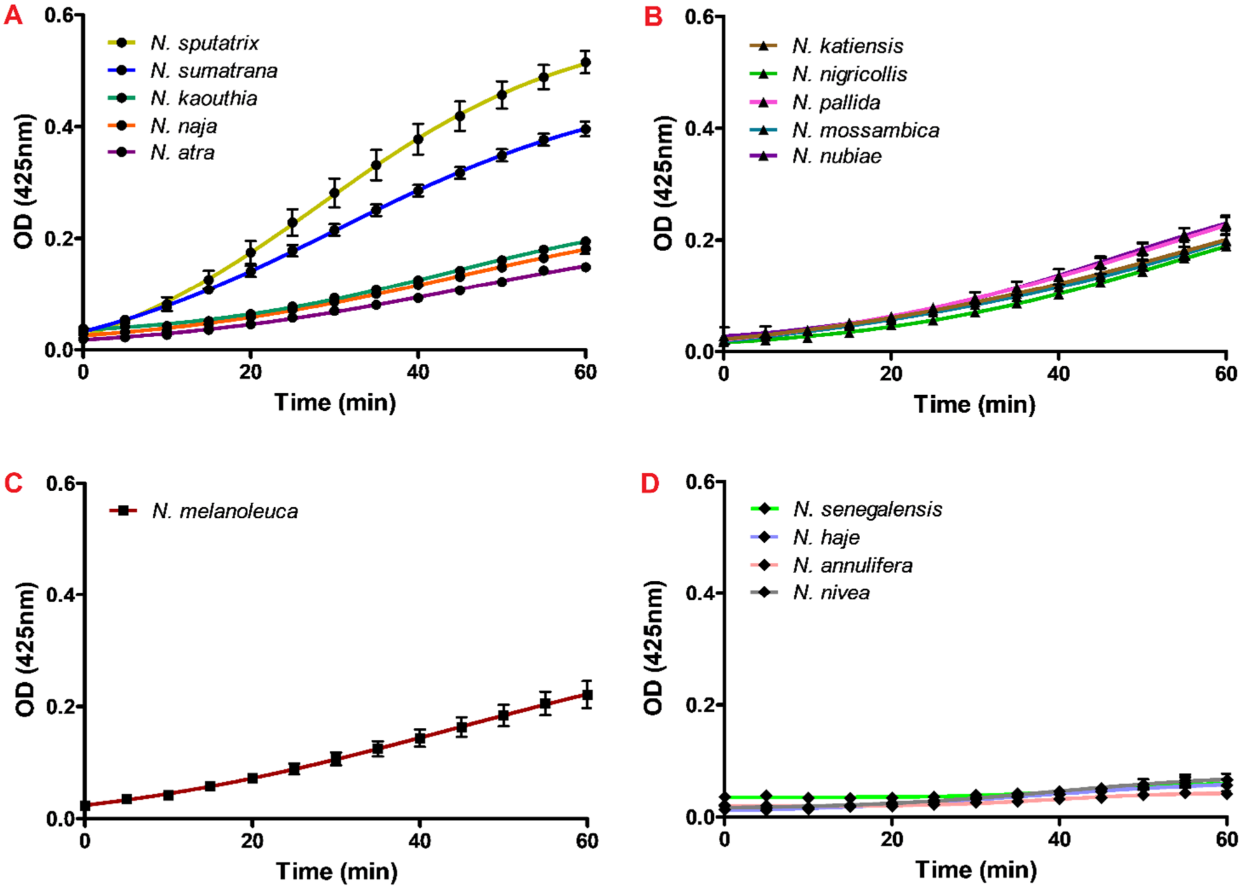

Figure S2. Time-dependent absorbance changes in colorimetric assay for the venoms of four subgenera of cobra. (**A**) *Naja*, (**B**) *Afronaja*, (**C**) *Boulengerina*, and (**D**) *Uraeus*. Changes in absorbance were due to the hydrolysis of the synthetic chromogenic substrate (NOBA), corresponding to the enzymatic activity of phospholipases A_2 in the venoms.

Table S1. Relative abundances of snake venom phospholipase A2 of 12 cobra species (Genus: *Naja*).

Subgenus of *Naja*	Cobra Species	Source	Relative Abundance of PLA2 (%)	Method of Protein Identification	References
Naja	*Naja naja*	Latoxan (Pakistan)	14.24	Bottom up proteomic: RP-HPLC, in-gel digestion, MALDI TOF/TOF and nano-ESI-LCMS/MS<br>Abundance calculation: gel densitometry x peak area under curve of chromatographic fraction	[1]
	Naja kaouthia	Pooled venom from Thailand	13.5	Bottom up proteomic: RP-HPLC, in-gel digestion, MALDI TOF/TOF and Orbitrap fusion mass spectrometry<br>Abundance calculation: gel densitometry x peak area under curve of chromatographic fraction	[2]
	Naja sputatrix	Latoxan (Indonesia)	31.24	Bottom up proteomic: RP-HPLC, in-solution digestion, nano-ESI-LCMS/MS<br>Abundance calculation: mean spectral intensity x peak area under curve of chromatographic fraction	[3]
	Naja atra	Venom from CDC, Taiwan	14.43	Bottom up proteomic: RP-HPLC, in-solution digestion, nano-ESI-LCMS/MS<br>Abundance calculation: mean spectral intensity x peak area under curve of chromatographic fraction	[4]
	Naja sumatrana	Pooled venom from Malaysia	32.3	Bottom up proteomic: RP-HPLC, in-solution digestion, nano-ESI-LCMS/MS<br>Abundance calculation: mean spectral intensity x peak area under curve of chromatographic fraction	[5]
Afronaja	*Naja nigricollis*	Pooled venom from Nigeria	21.9	Bottom up proteomic: RP-HPLC, N-terminal sequencing, SDS-PAGE, in-gel digestion, ESI-MS<br>Abundance calculation: gel densitometry x peak area under curve of chromatographic fraction	[6]
	Naja pallida	Latoxan (Kenya)	30.1	Bottom up proteomic: RP-HPLC, N-terminal sequencing, SDS-PAGE, in-gel digestion, ESI-MS<br>Abundance calculation: gel densitometry x peak area under curve of chromatographic fraction	
	Naja nubiae	Latoxan North Africa)	26.4	Bottom up proteomic: RP-HPLC, N-terminal sequencing, SDS-PAGE, in-gel digestion, ESI-MS<br>Abundance calculation: gel densitometry x peak area under curve of chromatographic fraction	
	Naja mossambica	Latoxan (Tanzania)	27.1	Bottom up proteomic: RP-HPLC, N-terminal sequencing, SDS-PAGE, in-gel digestion, ESI-MS<br>Abundance calculation: gel densitometry x peak area under curve of chromatographic fraction	
	Naja katiensis	Latoxan (Burkina Faso)	29.0	Bottom up proteomic: RP-HPLC, N-terminal sequencing, SDS-PAGE, in-gel digestion, ESI-MS<br>Abundance calculation: gel densitometry x peak area under curve of chromatographic fraction	
Boulengerina	*Naja melanoleuca*	Latoxan (Uganda)	12.9	Bottom up proteomic: RP-HPLC, in-gel digestion, MALDI TOF/TOF<br>Abundance calculation: gel densitometry x peak area under curve of chromatographic fraction	[7]
Uraeus	*Naja haje*	Pooled venom from Morocco	4	Bottom up proteomic: RP-HPLC, in-solution digestion, nano-ESI-LCMS/MS	[8]

Reference

1. Wong, K.Y.; Tan, C.H.; Tan, K.Y.; Quraishi, N.H.; Tan, N.H. Elucidating the biogeographical variation of the venom of *Naja naja* (spectacled cobra) from Pakistan through a venom-decomplexing proteomic study. *J Proteomics* **2018**, *175*, 156–173, doi:10.1016/j.jprot.2017.12.012.
2. Tan, K.Y.; Tan, C.H.; Fung, S.Y.; Tan, N.H. Venomics, lethality and neutralization of *Naja kaouthia* (monocled cobra) venoms from three different geographical regions of Southeast Asia. *J Proteomics* **2015**, *120*, 105–125, doi:10.1016/j.jprot.2015.02.012.
3. Tan, N.H.; Wong, K.Y.; Tan, C.H. Venomics of *Naja sputatrix*, the Javan spitting cobra: A short neurotoxin-driven venom needing improved antivenom neutralization. *J Proteomics* **2017**, *157*, 18–32.
4. Liu, C.-C.; You, C.-H.; Wang, P.-J.; Yu, J.-S.; Huang, G.-J.; Liu, C.-H.; Hsieh, W.-C.; Lin, C.-C. Analysis of the efficacy of Taiwanese freeze-dried neurotoxic antivenom against *Naja kaouthia*, *Naja siamensis* and *Ophiophagus hannah* through proteomics and animal model approaches. *PLOS Negl. Trop. Dis.* **2017**, *11*, e0006138, doi:10.1371/journal.pntd.0006138.
5. Yap, M.K.; Fung, S.Y.; Tan, K.Y.; Tan, N.H. Proteomic characterization of venom of the medically important Southeast Asian *Naja sumatrana* (Equatorial spitting cobra). *Acta tropica* **2014**, *133*, 15–25, doi:10.1016/j.actatropica.2014.01.014.
6. Petras, D.; Sanz, L.; Segura, Á.; Herrera, M.; Villalta, M.; Solano, D.; Vargas, M.; León, G.; Warrell, D.A.; Theakston, R.D.G., et al. Snake Venomics of African spitting cobras: Toxin composition and assessment of congeneric cross-reactivity of the Pan-African EchiTAb-Plus-ICP Antivenom by antivenomics and neutralization approaches. *J. Proteome Res.* **2011**, *10*, 1266–1280, doi:10.1021/pr101040f.
7. Lauridsen, L.P.; Laustsen, A.H.; Lomonte, B.; Gutierrez, J.M. Exploring the venom of the forest cobra snake: Toxicovenomics and antivenom profiling of *Naja melanoleuca*. *J Proteomics* **2017**, *150*, 98–108, doi:10.1016/j.jprot.2016.08.024.
8. Malih, I.; Ahmad rusmili, M.R.; Tee, T.Y.; Saile, R.; Ghalim, N.; Othman, I. Proteomic analysis of Moroccan cobra Naja haje legionis venom using tandem mass spectrometry. *J Proteomics* **2014**, *96*, 240–252, doi:http://dx.doi.org/10.1016/j.jprot.2013.11.012.
